# Diversity of the Germination Apparatus in *Clostridium botulinum* Groups I, II, III, and IV

**DOI:** 10.3389/fmicb.2016.01702

**Published:** 2016-10-28

**Authors:** Jason Brunt, Arnoud H. M. van Vliet, Fédor van den Bos, Andrew T. Carter, Michael W. Peck

**Affiliations:** ^1^Gut Health and Food Safety, Institute of Food ResearchNorwich, UK; ^2^School of Veterinary Medicine, Faculty of Health and Medical Sciences, University of SurreyGuildford, UK

**Keywords:** *C. botulinum*, C. sporogenes, spore, germination, germinant receptors, cortex-lytic enzymes

## Abstract

*Clostridium botulinum* is a highly dangerous pathogen that forms very resistant endospores that are ubiquitous in the environment, and which, under favorable conditions germinate to produce vegetative cells that multiply and form the exceptionally potent botulinum neurotoxin. To improve the control of botulinum neurotoxin-forming clostridia, it is important to understand the mechanisms involved in spore germination. Here we present models for spore germination in *C. botulinum* based on comparative genomics analyses, with *C. botulinum* Groups I and III sharing similar pathways, which differ from those proposed for *C. botulinum* Groups II and IV. All spores germinate in response to amino acids interacting with a germinant receptor, with four types of germinant receptor identified [encoded by various combinations of *gerA, gerB*, and *gerC* genes (*gerX*)]. There are three gene clusters with an ABC-like configuration; ABC [*gerX1*], ABABCB [*gerX2*] and ACxBBB [*gerX4*], and a single CA-B [*gerX3*] gene cluster. Subtypes have been identified for most germinant receptor types, and the individual GerX subunits of each cluster show similar grouping in phylogenetic trees. *C. botulinum* Group I contained the largest variety of *gerX* subtypes, with three *gerX1*, three *gerX2*, and one *gerX3* subtypes, while *C. botulinum* Group III contained two *gerX1* types and one *gerX4*. *C. botulinum* Groups II and IV contained a single germinant receptor, *gerX3* and *gerX1*, respectively. It is likely that all four *C. botulinum* Groups include a SpoVA channel involved in dipicolinic acid release. The cortex-lytic enzymes present in *C. botulinum* Groups I and III appear to be CwlJ and SleB, while in *C. botulinum* Groups II and IV, SleC appears to be important.

## Introduction

All strains of *Clostridium botulinum* form the highly potent botulinum neurotoxin, the agent responsible for botulism, a severe and often fatal neuroparalytic disease of humans and animals (Hatheway, 1988; Hauschild, 1989; Lindström et al., 2009; Peck, 2009; Peck et al., 2011; Johnson, 2013; Carter and Peck, 2015). There are seven confirmed botulinum neurotoxins (types A to G), and approximately forty different subtypes (Carter and Peck, 2015; Hill et al., 2015; Williamson et al., 2016). The botulinum neurotoxin is the most powerful toxin known, with as little as 30–100 ng sufficient to cause human botulism. Humans are susceptible to three distinct types of botulism. Foodborne botulism is an intoxication associated with consumption of botulinum neurotoxin preformed in food. Infant/intestinal (adult) botulism is an infection associated with growth and neurotoxin formation in the infant gut, while wound botulism is an infection associated with growth and neurotoxin formation in a wound (often following drug abuse). In humans, symptoms of botulism typically commence with blurred vision, followed by an acute symmetrical descending bilateral paralysis, and eventually paralysis of the respiratory/cardiac muscles (Hatheway, 1988; Hauschild, 1989; Lindström et al., 2009; Peck, 2009; Peck et al., 2011; Johnson, 2013; Carter and Peck, 2015).

All strains of *C. botulinum* also form highly resistant endospores that are ubiquitous in the environment and may contaminate foods (Dodds, 1992; Carlin et al., 2004; Peck, 2010; Peck et al., 2010; Barker et al., 2016) and which, under favorable conditions germinate to produce vegetative cells that multiply and form neurotoxin. Spore germination is commonly initiated by a germinant receptor (GR) responding to nutrient germinants, and is followed by the release of dipicolinic acid (DPA) and partial core hydration. Next, cortex-lytic enzymes (CLEs) degrade the spore cortex, permitting additional core hydration and core expansion. Since spore germination is the key stage in the transition from dormant spore to vegetative cell, a greater understanding of the mechanisms involved in this process may contribute to an improved control of *C. botulinum*. Spore germination is relatively well understood in *Bacillus* (Setlow, 2014), and significant developments have been recently made in various species of *Clostridium*, including *C. botulinum* (Broussolle et al., 2002; Alberto et al., 2003; Paredes-Sabja et al., 2008b, 2009a; Burns et al., 2010; Adam et al., 2011; Banawas et al., 2013; Brunt et al., 2014, 2015; Meaney et al., 2015; Kevorkian et al., 2016).

*Clostridium botulinum* is not a homogeneous species, but a collection of four discrete bacterial groups (*C. botulinum* Groups I–IV) that share the common feature of forming botulinum neurotoxin. While all are obligately anaerobic bacteria, the four groups are sufficiently distinct to merit allocation to individual species. For each *C. botulinum* group, closely related non-toxigenic bacteria are known. *C. botulinum* Group I (proteolytic *C. botulinum*) is a major cause of botulism in humans (foodborne, infant, and wound), and strains form one or more neurotoxins of types A, B, or F (Hatheway, 1988; Peck et al., 2011; Johnson, 2013). *C. botulinum* Group I is a highly proteolytic and mesophilic bacterium that forms very heat resistant spores. The “Botulinum cook” (121°C/3 min) given to low acid canned foods is designed to inactivate these spores (Peck, 2009). *C. sporogenes* and *C. botulinum* Group I are closely related bacteria (Collins et al., 1994; Sebaihia et al., 2007; Jacobson et al., 2008; Carter et al., 2009; Bradbury et al., 2012). *C. sporogenes* is a significant cause of food spoilage (McClure, 2006), and due to its close physiological similarity to *C. botulinum* Group I is widely used as a surrogate in demonstrating the effectiveness of food preservation processes (Brown et al., 2012; Taylor et al., 2013). Recent analysis indicates that several strains that form type B neurotoxin, and were previously classified as *C. botulinum* Group I, appear more like strains of *C. sporogenes* that have acquired a neurotoxin gene (Carter et al., 2009; Weigand et al., 2015; Williamson et al., 2016). *C. botulinum* Group II (non-proteolytic *C. botulinum*) is an important cause of foodborne botulism in humans, and is a concern for the safe production of minimally heat-processed refrigerated foods (Peck and Stringer, 2005; Peck, 2006; Peck et al., 2008). Strains form a single neurotoxin of type B, E, or F. *C. botulinum* Group II is a saccharolytic and psychrotrophic bacterium that forms spores of moderate heat resistance (Peck, 2009; Stringer et al., 2013). Strains of *C. botulinum* Group III form a single neurotoxin of type C or type D or more commonly a hybrid neurotoxin that comprises elements of each (type C/D or D/C), and are responsible for botulism in a wide range of animal species (Lindstrom et al., 2004; Sharpe et al., 2008; Woudstra et al., 2015). *C. botulinum* Group III is a saccharolytic and mesophilic bacterium that closely resembles *C. novyi* and *C. haemolyticum* (Skarin and Segerman, 2014) and forms spores of high heat resistance (Sasaki et al., 2001; Woudstra et al., 2015). *C. botulinum* Group IV (also known as *C. argentinense*) is the least studied *C. botulinum* group. This proteolytic and mesophilic bacterium forms type G neurotoxin and spores of high heat resistance. While experiments have shown its type G toxin to be toxigenic in animals, it has been weakly associated with botulism cases (Peck, 2009). Closely related non-toxigenic bacteria include *C. subterminale*.

The rapid technical development in genome sequencing, as well as the reductions in cost, now allow for comparative genomics of large collections of bacteria. In the present study, genome sequences have been used to establish the spore germination pathways, from germination receptors to spore-cortex-lytic hydrolases, for *C. botulinum* Groups I–IV. These bioinformatic approaches have been coupled to experimental analysis of germination stimuli. *C. botulinum* spores were found to contain GRs that responded to amino acids, and CLEs that resembled those described in other species. Spore germination appeared similar in *C. botulinum* Groups I and III, with subtle differences to that in *C. botulinum* Groups II and IV.

## Materials and Methods

### Genome Sequences Included in This Study

Genome sequences were downloaded in FASTA file format as contigs or complete genome sequences from the NCBI website^1^. Supplementary Table S1 contains GenBank/EMBL/DDBJ accession numbers of each genome sequence used, as well as assignment to *C. botulinum* Groups I–IV. The *C. botulinum* Group I data set comprised 92 *C. botulinum* and 8 *C. sporogenes* genome sequences, while those for *C. botulinum* Groups II and III comprised 24 and 31 *C. botulinum* genome sequences, respectively. A single *C. botulinum* Group IV genome was also included.

### Phylogenetic Analyses

A combined phylogenetic tree of all 156 genome sequences was generated using Feature Frequency Profiling with FFPry version 3.19^2^ (Sims et al., 2009) with a word length of *L* = 18 as described in (van Vliet and Kusters, 2015). The program FFPboot was used for bootstrap analysis of the tree generated, using the default settings, and run for 100 replicates. Individual phylogenetic trees of *C. botulinum* Groups I–III were generated from core genome single nucleotide polymorphisms (SNPs), identified using the parSNP version 1.2 program from the Harvest suite (Treangen et al., 2014) with the “-a 13 -c -x” switches, with bootstrap values provided by the ParSNP output.

### Comparative Genomics of GerX and Cortex-Lytic Enzymes

**FIGURE 1:**
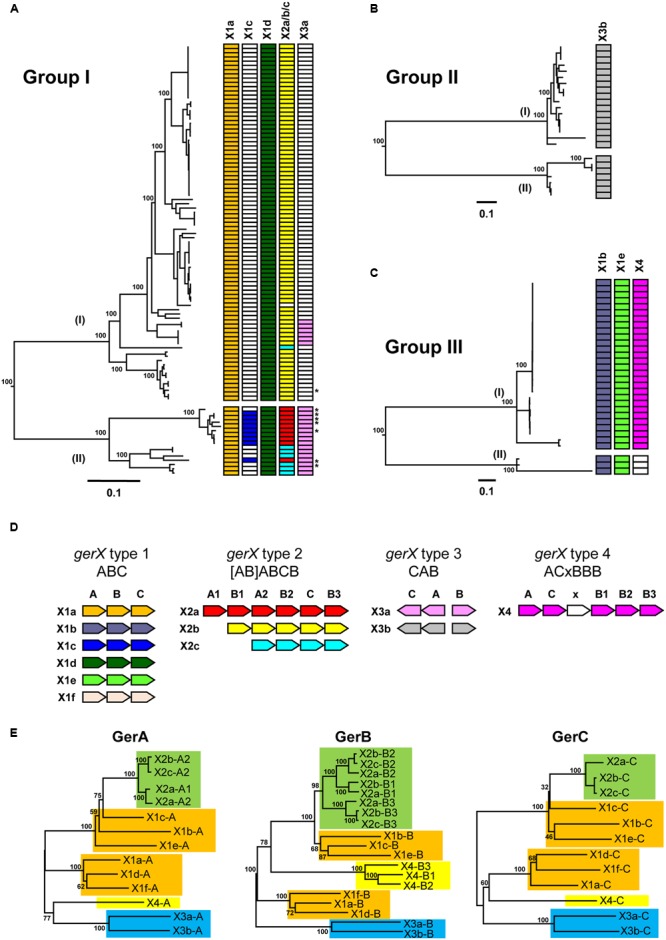
**Distribution of GerX receptor gene clusters in *Clostridium botulinum* Groups I to IV and *C. sporogenes* genomes. (A)** Distribution of *gerX* types in *C. botulinum* Group I and *C. sporogenes* genomes. The asterisks represent *C. sporogenes* strains. The colors in trees **(A–C)** correspond with the colors attributed to each individual gerX subtype shown in **(D)**. White blocks represent absence of the specific germinant receptor subtype. **(B)** Distribution of *gerX* types in *C. botulinum* Group II genomes. **(C)** Distribution of *gerX* types in *C. botulinum* Group III genomes. A full list of isolates included and their *gerX* gene clusters is given in Supplementary Table S4. The phylogenetic trees in (A–C) are based on single nucleotide polymorphisms (SNPs) as determined using ParSNP (Treangen et al., 2014). Values shown at branches represent bootstrap values provided by ParSNP. **(D)** Genetic organization of the *gerX* subtypes. Type 1 represents the *gerABC* layout, Type 2 represents the (AB) ABCB layout, Type 3 the CA-B layout as a bicistronic gene cluster, and Type 4 an ACxBBB gene cluster. Subtype *gerX1f* is only found in *C. botulinum* Group IV which is not shown in **(A–C)**. **(E)** Phylogenetic tree of Ger proteins. Representative reference genomes used are given in Supplementary Table S2. Colors represent the clustering based on GerX type. GerX type 1 is highlighted in gold, GerX type 2 in green, GerX type 3 in blue, GerX type 4 in yellow. The GerA, GerB, and GerC amino acid sequences each cluster into different subtypes. GerA, GerB, and GerC amino acid sequences were aligned with MEGA v 7.0, followed by creation of a phylogenetic tree using the Neighbor joining algorithm. Values shown at branches represent bootstrap values from 100 replicates.

Genome sequences were provisionally annotated using Prokka version 1.12 (Seemann, 2014) and used for comparative genomics using Roary version 3.5.7 (Page et al., 2015). The *gerX* clusters and associated upstream and downstream genes were extracted from the comparisons to assess conservation of the genomic organization of the *gerX* clusters, as outlined in **Figure 1D** and Supplementary Table S2. Alignments were made with MEGA version 6.0 (Tamura et al., 2011), and used for generation of phylogenetic trees using the Neighbor Joining option. Figtree^3^ was used for annotation of phylogenetic trees. Genome sequences were genotyped for the different *gerX* clusters by *in silico* hybridisation using 60 nt oligonucleotides from the different *gerX* gene clusters, using the Microbial In Silico Typing (MIST) software package (Kruczkiewicz et al., 2013) and the NCBI Blast+ version 2.28 executables. Representative gene numbers for the *gerX* clusters and CLE genes from reference genomes are given in Supplementary Table S2. BLAST searches of the Prokka-annotated genomes were used to assess the level of variation between predicted GerX and CLE proteins encoded by the genomes. All generated data and phylogenetic trees are available in Supplementary Data Sheet 1.

### Protein Bioinformatics

Relevant predicted amino acid sequences of the germination proteins of interest were imported into the Geneious 8.1.7 software (Biomatters^4^) package from NCBI. Signal cleavage sites were predicted using sigcleave, which is part of the EMBOSS suite (Rice et al., 2000). Protein domain analysis was performed using Pfam (Punta et al., 2012) and InterProScan to annotate proteins with families and domains (Quevillon et al., 2005). Transmembrane helices were characterized using Transmembrane Hidden Markov models (TMHMM; Krogh et al., 2001). Protein structure predictions were also analyzed using the PSIPRED Protein Sequence Analysis Workbench (Buchan et al., 2013).

### Bacterial Strains and Growth Conditions

*Clostridium sporogenes* strain ATCC15579 was grown at 37°C in anaerobic tryptone-yeast-glucose medium (TYG). *Escherichia coli* strain Top10 (Invitrogen) was used for plasmid maintenance and the *E. coli* strain CA434 (Purdy et al., 2002) was used for conjugal transfer. *E. coli* strains were grown aerobically in Luria–Bertani medium (LB) at 37°C. Where appropriate, growth medium was supplemented with antibiotics at the following final concentrations; chloramphenicol 25 μg/ml, cycloserine 250 μg/ml, thiamphenicol 15 μg/ml, erythromycin 2.5 μg/ml, and the chromogenic substrate 5-bromo-4-chloro-3-indolyl-β-D-galactopyranoside (X-Gal) 80 μg/ml. All bacterial media supplements were purchased from Sigma.

### PCR and Cloning

Constructed mutants and plasmids utilized in this study are presented in Supplementary Table S3. Primers used for verification of successful insertion events are also listed in Supplementary Table S3. PCR experiments were performed using Phusion High-Fidelity PCR Master Mix with GC Buffer kit (Thermo Fisher). Plasmid isolation and PCR purification was performed using the Wizard *Plus* SV Minipreps DNA Purification System and the Wizard SV Gel and PCR Clean-Up System (Promega) respectively, as defined in the provided Technical Manual. Chromosomal DNA isolation from potential mutants was prepared as previously described (Sebaihia et al., 2007).

### Generation and Characterisation of *spoVA* Mutants and Their Complements

Mutants of *C. sporogenes* strain ATCC15579 were generated using the Clostron system as previously described (Brunt et al., 2014). Briefly, target sites were identified using the Perutka method (Perutka et al., 2004) and mutants were generated (Supplementary Table S3) as described (Heap et al., 2010). Re targeted introns were synthesized and ligated into the pMTL007C-E2 vector by DNA 2.0 (Menlo Park, USA). Retargeted intron plasmids were transformed into *E. coli* CA434. Confirmed (sequenced) plasmids were then transferred by conjugation into their respective clostridial host. For mutant complementation, plasmid pMTL83151 was used (Heap et al., 2009). Primers bearing restriction sites compatible with pMTL83151 (*Bam*HI and *Nhe*I) were used to amplify the *spoVA* gene cluster and its 5′ non-coding region, covering the predicted putative promoter. The resulting PCR product was digested with *Bam*HI and *Nhe*I before being ligated into the pMTL83151 plasmid. Following confirmation by sequencing, complementation plasmids were transconjugated into their respective mutants using *E. coli* CA434 as described previously. The capacity of *C. sporogenes spoVA* mutants to form spores was assessed following incubation in anaerobic TYG broth at 37°C for 72 h. Spore formation was visualized every 24 h, in at least twenty fields, using phase-contrast microscopy. The number of heat resistant spores formed after 72 h was determined by heating the culture (80°C, 15 min), serial dilution in 0.85% saline, and plating in triplicate on to TYG agar before incubation anaerobically (37°C, 72 hrs).

## Results and Discussion

### Core Genome SNP Analysis of *C. botulinum* Genomes Confirms Clustering into Four Distinct Groups

To investigate the relationship between germination genes and genome phylogeny, we obtained 156 *C. botulinum* genome sequences. These were first clustered using feature frequency profiling (Sims et al., 2009; van Vliet and Kusters, 2015), to obtain an overview of the phylogenetic relationships within these 156 genome sequences. This initial analysis showed that the 156 genomes separated into four distinct phylogenetic groups (Supplementary Figure S1), thus giving 100 *C. botulinum* Group I (including *C. sporogenes*), 24 *C. botulinum* Group II, 31 *C. botulinum* Group III, and 1 *C. botulinum* Group IV genomes. The phylogenetic relationships within each group were determined using core genome SNP analysis, highlighting that Groups I–III each consisted of two distinct clusters of isolates, thus confirming the heterogeneity of the *C. botulinum* species. All but one of the *C. sporogenes* genomes clustered in *C. botulinum* Group I (lineage II), together with nine *C. botulinum* genomes forming a more distantly related lineage (**Figure 1A**, lineage II and Supplementary Table S4). It was recently reported that *C. botulinum* strains CDC68016, ATCC 51387, Prevot 1662, Osaka05, and Okayama2011 were defined as *C. sporogenes*-like strains that may have acquired a neurotoxin gene via horizontal transfer of plasmid DNA (Weigand et al., 2015). Conversely, *C. sporogenes* strain CDC24533 belonging to lineage I is a *C. botulinum* Group I strain that probably lost its neurotoxigenic plasmid (Weigand et al., 2015). *C. botulinum* Group II strains were separated into two distinct lineages (**Figure 1B**; Supplementary Table S4). Lineage I comprises BoNT/E producing strains only, and while lineage II is dominated by BoNT/B producing strains some BoNT/E and /F producing strains are also present, confirming a previous microarray study (Stringer et al., 2013). Members of *C. botulinum* Group III also split into two distinct lineages (**Figure 1C**; Supplementary Table S4). Finally, a single strain of *C. botulinum* Group IV was examined. In summary, our phylogenetic analysis agrees with previous work that the lack of phylogenetic relationship between the Groups is sufficient to consider each Group as a separate species.

### Germinant Receptor Subunits May Have Co-evolved and Could Allow Adaptation to New Environmental Niches

Under suitable conditions, the dormancy of bacterial spores is broken, and germination occurs. This is often initiated by a GR located in the spore inner membrane responding to nutrient germinants, followed by the release of DPA and partial core hydration. The *C. botulinum* and *C. sporogenes* GR is generally composed of three proteins (GerA, GerB, and GerC) that are encoded by their respective *ger* genes in a multi gene locus (Brunt et al., 2014). The designation, *gerX* implies that the cognate germinant molecule for the receptor encoded by this gene cluster is unknown. All 156 *C. botulinum* and *C. sporogenes* genome sequences were interrogated for *gerX* clusters and their flanking genes to assess conservation of the *gerX* cluster genomic organization. Four different types of *gerX* cluster were identified (**Figure 1D**). These different cluster types were further separated into subtypes based on similarity of DNA and predicted protein sequences of their *gerA, gerB*, and *gerC* genes, together with their genomic organization (**Figure 1D**). Analysis showed six subtypes displaying a monocistronic *gerABC* gene order (*gerX* type 1a–f), three subtypes that possess a *gerABCB* core, preceded by *gerAB, gerB* or nothing (*gerX* type 2a–c), two subtypes with a bicistronic divergent *gerCA* and *gerB* gene cluster (*gerX* type 3a–b), and one with a *gerACxBBB* configuration (*gerX* type 4; **Figure 1D**). Using BLASTP, the similarity within each GerX1 and GerX3 subtype was more than ca. 90% (except GerX1c ∼85%), while the similarity between each subtype was ca. 20–65%.

Comparison of the amino acid sequences of the respective GerA, GerB, and GerC proteins showed that these form clusters according to the GerX subtype (**Figure 1E**). For example, the GerA, GerB, and GerC proteins of the X2 type each clustered together, as do the GerA, GerB, and GerC components of the X1a, X1b and, X1f subtypes, and the X1c, X1d, and X1e subtypes. Interestingly, the three GerB proteins of the GerX4 type all clustered closely together, and separately from the other GerB proteins (**Figure 1E**). The fact that all three GerX subunits group together within these four distinct lineages may imply that individual genes within each cluster could have co-evolved, and that the different cluster subtypes may have not arisen through insertional events (except for type 2).

*gerX* type 1 is an archetypal tricistronic ABC configuration receptor, with six subtypes (**Figure 1D**). Each subtype is unique to a single *C. botulinum* Group. All *C. botulinum* Group I/*C. sporogenes* strains possess *gerX1a* and *gerX1d*, while several strains in *C. botulinum* Group I/*C. sporogenes* lineage II also possess *gerX1c* (**Figure 1A**). *gerX1b* and *gerX1e* are both present in all *C. botulinum* Group III strains (**Figure 1C**), and *gerX1f* is found in *C. botulinum* Group IV. *gerX1a* and *gerX1d* (but not *gerX1c*) are present in *C. botulinum* Group I strain ATCC 3502 and *C. sporogenes* ATCC 15579, and their functionality has been demonstrated (Brunt et al., 2014). The mutation of putative germination *gerXA* receptor genes revealed that both *gerX1s* were essential for amino acid stimulated germination in *C. botulinum* Group I strain ATCC 3502, while in *C. sporogenes* ATCC 15579 only *gerX1d* was essential for amino acid stimulated germination (Brunt et al., 2014). The fact that no obvious structural evidence could be found to explain the functional differences between this receptor in *C. botulinum* and *C. sporogenes* suggests that the answer may lie in subtle differences between their respective primary amino acid sequences. Interestingly, receptor types *gerX1a* (*C. botulinum* Group I/*C. sporogenes*), *gerX1b* (*C. botulinum* Group III), and *gerX1f* (*C. botulinum* Group IV), are flanked by homologs of a hypothetical protein immediately upstream of *gerXA* and a stage II sporulation protein immediately downstream of *gerXC*. This suggests a conserved location in *C. botulinum* Group I/*C. sporogenes*, and in *C. botulinum* Groups III and IV. However, the other slightly more distantly related *gerX1* subtypes (*gerX1c, gerX1d*, and *gerX1e*; **Figure 1E**) are not immediately flanked by the same genes.

The GerXA, GerXB, and GerXC proteins present in GerX type 2 each form a distinct clade separate from other GR proteins (and are shaded green in **Figure 1E**). GerX type 2 is exclusively possessed by strains of *C. botulinum* Group I and *C. sporogenes*, and all strains carry a single version of this larger GR gene cluster except for strain 20497 (**Figure 1A**; Supplementary Table S4). Further examination of the genome of strain 20497 reveals a ∼77 kb deletion compared to the closely related strain 20427, and this ∼77 kb region includes this putative GR gene cluster. The genes encoding the GerX type 2 proteins appear to be in a stable genomic environment, as the GR gene clusters always bear on their 5′ flank an alanine racemase CDS and on their 3′ flank a small conserved hypothetical protein CDS. Alanine racemase is able to convert the germinant L-alanine into the germination inhibitor D-alanine in *B. cereus* (Dodatko et al., 2009). This locus is conceivably a ‘transferable plasticity region’ as the configuration and number of GerX CDSs appears to be interchangeable, implying that this region may serve as a recombinational hot-spot for the *gerX* subunits. In addition, a small fragment of a *gerXA* gene apparently was often inserted into the 5′ end of the first ‘extra’ *gerXB* gene of *C. botulinum* Group I; one example of which has been recently described in some detail (Brunt et al., 2014). The functionality of two of these gene clusters (*gerX2b* and *gerX2c* configurations) has recently been tested in *C. botulinum* Group I strain ATCC 3502 and *C. sporogenes* ATCC 15579 (Brunt et al., 2014). Mutagenesis of these multi gene loci revealed that neither was able to promote germination alone, although *gerX2c* formed part of a complex involved in controlling the germination rate in *C. sporogenes* (Brunt et al., 2014). Moreover, it is conceivable that they could individually respond to some other as yet unknown germinant or environmental niche. It is perhaps pertinent to consider why these putative GR gene clusters vary in their configuration and in particular have additional *gerXB* genes (except for *gerX2a* which has an additional *gerXA*). Why does this not appear to be the case with *gerXA* or *gerXC* genes? Furthermore, the genomes of all *C. botulinum* Group I strains also contain a gene encoding an orphan *gerB* subunit. Although a monocistronic *gerA* gene subunit has been shown to be functional in *C. perfringens*, albeit only having a minor role (Paredes-Sabja et al., 2008b; Banawas et al., 2013), there is no current evidence of a functioning monocistronic *gerB* gene in the genus *Clostridium*. From our bioinformatics analysis the GerXB protein subunits are up to 457 residues in length and consist of 7–10 transmembrane helices. The GerXB proteins belong to the superfamily of membrane-associated single-component membrane transporters (Setlow, 2014; Moir and Cooper, 2016). However, this homology is largely based on structure as the sequence homology is low. Current evidence also suggests that the GerB protein is responsible for germinant binding (Christie et al., 2008; Christie et al., 2010) and may stabilize and/or influence the quantity of GerC proteins (Cooper and Moir, 2011). Perhaps one attractive proposal is that if the GerXB protein does indeed contain the germinant binding site, then the ability of strains to swap GerXB units or to possess multiple GerXB units may enable them to adapt to exploit new environmental niches.

GerX type 3 is present in some *C. botulinum* Group I lineage (I) strains and all lineage (II) strains (*gerX3a*), and in all *C. botulinum* Group II strains (*gerX3b*; **Figures 1A,B**). It is the only complete GR present in *C. botulinum* Group II, with the caveat that there are currently fewer sequenced *C. botulinum* Group II genomes available for analysis than there are *C. botulinum* Group I genomes. Analysis of this locus revealed an organization that is quite different to the ‘classical’ receptor organization with a GerXA CDS in the middle of the receptor (CA-B; **Figure 1D**), and a bicistronic transcriptional organization with *gerAC* and *gerB* genes in the opposite orientation. The organization of this receptor gene cluster is similar to the single one also observed in *C. perfringens* where the *gerK* locus includes a monocistronic *gerKB* in an orientation opposite to that of a bicistronic *gerKA-gerKC* (Paredes-Sabja et al., 2008b; Banawas et al., 2013). Furthermore, it has been demonstrated that the GerKC protein is the main GR protein involved in nutrient and non-nutrient germination (Banawas et al., 2013). Although no function has yet been formally assigned to this *ger* gene cluster, the fact that amino acid induced spore germination of three *C. botulinum* Group II strains has been previously reported makes it the prime candidate for encoding a functional germination receptor (Plowman and Peck, 2002). The GerXA, GerXB, and GerXC proteins present in GerX type 3 each form a separate clade from other *C. botulinum* GR proteins (shaded blue in **Figure 1E**).

There is a single example of GerX type 4, the receptor ACxBBB from some strains of *C. botulinum* Group III (**Figures 1C,D**). All three GerXB subunits clustered together [and are shaded yellow (**Figure 1E**)], and while similar to each other were more distantly related to other GerXB proteins (**Figure 1E**). GerXA and GerXC from GerX type 4 were also distinct from other related GR proteins (**Figure 1E**). The GerX(x) putative subunit, located in the middle of *C. botulinum* Group III germination cluster is a hypothetical protein of 74 residues and comprises a non-cytoplasmic domain (ca. 22 residues) at the N-terminus and a single transmembrane region (ca. 23 residues) in the centre. The protein is also predicted to have its own ribosome binding site and a predicted signal peptide; evidence that it is probably expressed. It also contains the ribosome binding site for the next gene (*gerXB*) in this locus. The amino acid sequences of GerX(x) revealed no homology to any other proteins outside of *C. botulinum* Group III. However, unrelated small (ca. 75 residues) proteins in or adjacent to other GR clusters have been described previously (Paredes-Sabja et al., 2011; Ramirez-Peralta et al., 2013). The functionality of GerX(x) remains to be tested but it is tempting to speculate that as this protein is only present in *C. botulinum* Group III, a pathogen strongly associated with animal botulism, this putative receptor subunit may be associated with a particular environmental niche.

### Spores Respond to a Diverse Range of Germinants

Germination of *Clostridium* spores is usually initiated by germinants that include amino acids and sugars (**Table 1**), and often proceeds more slowly than that observed with *Bacillus* (Brunt et al., 2014). Spore germination in *C. botulinum* Group I and *C. sporogenes* is triggered by a variety of amino acids, often in combination with L-lactate; although previous studies have described a variable effect of L-lactate (Broussolle et al., 2002; Alberto et al., 2003; Peck, 2009). A literature review of germination characteristics of *C. botulinum* Group I reveals that although most strains germinate with the addition of L-alanine, germination responses are strain, pH, temperature, and buffer dependent (Broussolle et al., 2002; Alberto et al., 2003; Peck, 2009; Brunt et al., 2014). Recently, analysis of *C. botulinum* strain ATCC 3502 revealed that GerX1a and GerX1d responded to a variety of amino acids and that they act in synergy and cannot function individually. *C. sporogenes* strain ATCC15579 also responded to a variety of amino acids. Although, only GerX1d was essential, GerX3a and GerX2c GRs form part of a complex involved in controlling the rate of amino acid stimulated germination (Brunt et al., 2014).

**Table 1:** Germinants of *Clostridium botulinum* Groups I–IV and *C. sporogenes* spores.

	Germinant	Reference
Group I	L-alanine, L-cysteine, L-methionine, L-serine, L-phenylalanine, glycine	Broussolle et al., 2002; Alberto et al., 2003; Brunt et al., 2014
*C. sporogenes*	L-alanine, L-cysteine, L-methionine, L-serine, L-phenylalanine,	Broussolle et al., 2002; Brunt et al., 2014
Group II	L-alanine, L-serine, L-cysteine, L-threonine, glycine	Ando and Iida, 1970; Ando, 1971; Plowman and Peck, 2002
Group III	No information	–
Group IV	L-cysteine, L-alanine^∗^	Takeshi et al., 1988

*L-lactate is often required as a co-germinant with the amino acids listed. ^∗^sub-optimal germination observed with *L*-alanine in *C. botulinum* Group IV.*

Spores of *C. botulinum* Group II also respond to a variety of amino acids (**Table 1**) (Ando and Iida, 1970; Ando, 1971; Plowman and Peck, 2002). In contrast to *C. botulinum* Group I, L-lactate is considered essential for amino acid induced germination in *C. botulinum* Group II (Plowman and Peck, 2002). The germination responses of *C. botulinum* Group II is dependent on strain, pH, temperature, and buffer (Ando and Iida, 1970; Ando, 1971; Plowman and Peck, 2002; Peck, 2009).

While various aspects of the physiology and genomics of *C. botulinum* Group III have been studied in some detail (Eklund and Dowell, 1987; Woudstra et al., 2015), relatively little is known about the properties of their spores or their spore germination characteristics. Based on analysis of the encoded GRs, it is anticipated that these spores will also respond to amino acid germinants. Current knowledge of spore germination in *C. botulinum* Group IV is also limited. However, one study has shown that spores optimally germinate with a mixture of L-cysteine, L-lactate, and bicarbonate and sub-optimally with L-alanine, L-lactate, and bicarbonate (Takeshi et al., 1988).

Observed inconsistencies in the results from different publications, together with our own experience, indicate that strain differences and the method used to produce spores, how they are maintained, and which buffer is used to evaluate germination, invariably has a direct effect on germination rates and extents.

### SpoVA is Implicated in Sporulation and DPA Release

*Clostridium* and *Bacillus* spores contain a large store of a 1:1 chelate of Ca^2+^ and pyridine-2, 6-dicarboxylic acid (DPA) within its core which contributes to spore dormancy and heat resistance (Paidhungat et al., 2000). It has been proposed that the SpoVA proteins are associated with the inner membrane and may form a mechanosensitive channel through which DPA may be transported, and thereby packaged during sporulation (Li et al., 2012; Korza and Setlow, 2013; Velasquez et al., 2014). One line of evidence supporting this hypothesis comes from work showing that SpoVAC can act as a non-selective solute channel when expressed in *E. coli* (Velasquez et al., 2014). There are three SpoVA proteins, and it is yet to be determined whether all three act together and/or interact with other proteins. For most spore-formers (including *C. perfringens*), DPA release precedes cortex hydrolysis during germination and is reported to also activate the CwlJ protein (Paidhungat et al., 2001). However, it has recently been found that in *Clostridium difficile* spore cortex hydrolysis precedes DPA release (Francis et al., 2015). It is presently unknown whether DPA release in *C. botulinum* and *C. sporogenes* follows that observed in *C. perfringens* or *C. difficile*. A role for SpoVA proteins in clostridial spore germination has been demonstrated in *C. perfringens* and *C. difficile* (Paredes-Sabja et al., 2008a; Donnelly et al., 2016).

**FIGURE 2:**
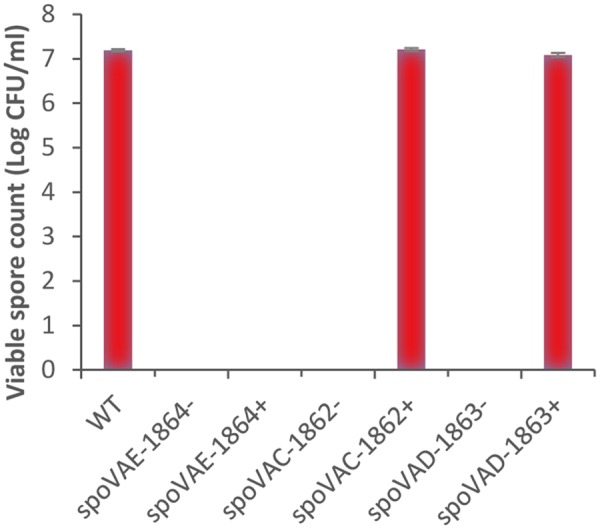
**Phenotypic analysis of the *spoVA* mutations in *C. sporogenes*.** *C. sporogenes* wild type and *spoVAC, spoVAD*, and *spoVAE* mutants were grown anaerobically at 37°C for 72 h, to allow spore formation. Samples were then heated (80°C, 15 min), serially diluted in 0.85% saline, and plated on to tryptone-yeast-glucose medium agar before incubation anaerobically (37°C, 72 h). Data presented represent the colony-forming units/ml from triplicate plates, with error bars representing the standard deviation of the mean.

In the present study, bioinformatics analysis revealed that strains of *C. botulinum* Groups I–III and *C. sporogenes* each contain one gene cluster encoding *spoVAC, spoVAD*, and *spoVAE*. In contrast the single strain of *C. botulinum* Group IV studied to date possesses two gene clusters encoding the SpoVA proteins. *C. botulinum* SpoVAC, a paralog of SpoVAE, is typically 153 residues long and contains three or four membrane-spanning regions. SpoVAE is approximately 118 residues long and also contains three or four membrane-spanning regions and a predicted signal peptide (*C. botulinum* Group III). SpoVAD is typically 333 residues long. SpoVAD has been shown to bind specifically to DPA and Ca-DPA (Li et al., 2012). To characterize the functionality of SpoVA proteins in *C. sporogenes* ATCC 15579, a series of single insertion mutants (*spoVAE*-1864^-^, *spoVAC*-1862^-^, *spoVAD*-1863^-^) were constructed and subsequently complemented (*spoVAE*-1864^+^, *spoVAC*-1862^+^, *spoVAD*-1863^+^). Phenotypic analysis of the *spoVA* mutations revealed that the products of all three genes were required for successful sporulation, and complementation restored similar wild type levels of sporulation, except for spoVAE-1864^-^ (**Figure 2**). Furthermore, attempts to complement the spoVAE-1864^-^ using a plasmid that contained the entire spoVA locus (i.e., *spoVAE, spoVAC, spoVAD*) also failed to complement spoVAE-1864^-^ mutant. The failure of some plasmid complemented mutants to regain wild type sporulation levels in clostridia has been reported previously (Li et al., 2011; Brunt et al., 2014; Meaney et al., 2015). Mutations in the *spoVA* operon of *B. subtilis* strains result in the lysis of immature spores during sporulation, most likely due to their lack of DPA (Tovar-Rojo et al., 2002). The cause of this lysis is unknown but it may be due in part to the CLE SleB (see below) which is activated in spores lacking DPA (Tovar-Rojo et al., 2002). Similarly, although we did initially observe occasional endospores produced by SpoVA mutants by phase microscopy, these endospores lysed during sporulation and none were observed at the end of the sporulation experiment. Furthermore, no heat resistant endospores were detected following heating (80°C, 15 min) and subsequent plating on microbiological growth medium. In contrast to our findings for *C. sporogenes* and those of *B. subtilis*, mutations of the *spoVA* operon in *C. perfringens* and *spoVAC in C. difficile* did not result in the lysis of immature spores and spores germinated relatively normally, but also had a high water content and decreased heat resistance (Paredes-Sabja et al., 2008a; Donnelly et al., 2016). Taken together, the sequence homology and phenotypic results presented here suggest that the SpoVA proteins may perform a similar role in *C. botulinum* and *C. sporogenes* to those reported in *Bacillus*, specifically the SpoVA proteins may be involved in DPA uptake during sporulation and DPA release during germination. Further studies are required, however, to confirm this assertion.

### Spore Cortex Hydrolysis and the Identification of Multiple Enzymes

In all *Bacillus* and *Clostridium* species [except *C. difficile* (Francis et al., 2015)], the release of DPA and various ions from the germinating spore is followed by the hydrolysis of the peptidoglycan in the spore cortex (Johnstone, 1994). The spore cortex peptidoglycan plays an important role in conserving spore dormancy and heat resistance (Atrih and Foster, 2001; Peck, 2009). Analysis of the spore cortex peptidoglycan reveals that its structure is highly conserved between *Bacillus* and *Clostridium* (Atrih and Foster, 2001). Cortex hydrolysis is reliant on activation of preformed CLEs that cleave the spore cortex peptidoglycan. Spores of *Bacillus* and some *Clostridium* species contain two redundant CLEs, CwlJ and SleB (Setlow et al., 2009; Paredes-Sabja et al., 2011; Meaney et al., 2015). CwlJ is activated by Ca^2+^-DPA release from the spore core (Paidhungat et al., 2001). The mechanism of SleB activation remains unknown, although YpeB is required for its localization and may be involved in regulating its activity (Li et al., 2013). *C. perfringens* and *C. difficile* have a solitary CLE, SleC, which is essential for peptidoglycan hydrolysis and germination (Miyata et al., 1995; Paredes-Sabja et al., 2009b; Burns et al., 2010). SleC is present as an inactive zymogen which becomes activated by cleavage of Pro-SleC by a Csp protease (Paredes-Sabja et al., 2009a).

**FIGURE 3:**
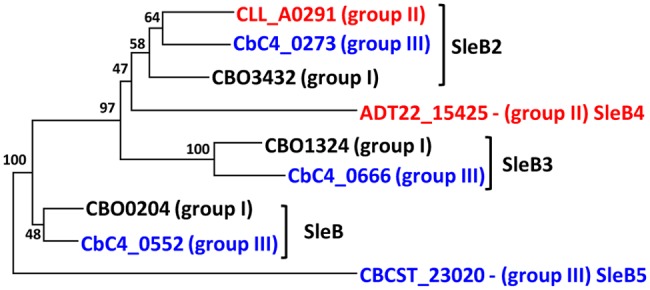
**Identification and naming of multiple SleB-like proteins.** SleB-like proteins were identified by homology searches in *C. botulinum* Groups I–III. Amino acid sequences were aligned with MEGA v 7.0, followed by creation of a phylogenetic tree using the Neighbor Joining algorithm. A full list of isolates included and their gene identifier is given in Supplementary Tables S2 and S4. Values shown at branches represent bootstrap values from 100 replicates.

**FIGURE 4:**
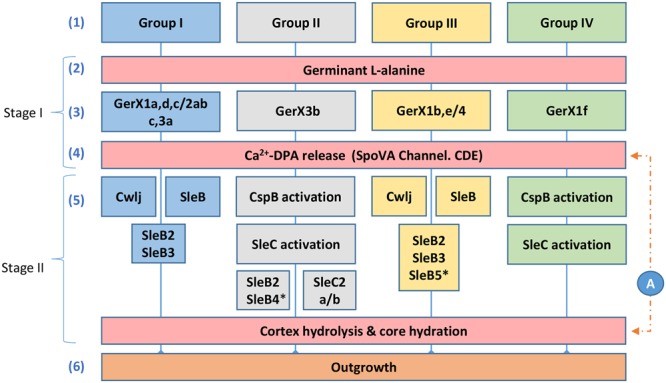
**Diagrammatic model for the proposed germination pathways of *C. botulinum* Groups I–IV.** One model for spore germination is proposed for *C. botulinum* Groups I and III, and a second related model is proposed for *C. botulinum* Groups II and IV. (1) *C. botulinum* Groups. Germination stage I; (2) and (3) germinant e.g., L-alanine binds to its cognate receptor located in the inner membrane (see **Figure 1**). (4) Ca^2+^-DPA is released through the SpoVA channel. Germination stage II; (5) Cortex-lytic enzymes (CLEs) are activated and then cleave the spore cortex peptidoglycan. *C. botulinum* Group I and Group III involves the CLEs CwlJ and SleB. *C. botulinum* Groups II and IV the CLE SleC is activated by CspB, followed by cortex hydrolysis. (6) Membrane and coat degradation, the recommencement of metabolism and eventually cell outgrowth of the newly formed cell. (A) The timing of the release of Ca^2+^-DPA is unknown in *C. botulinum* and may be released following germinant binding or after cortex hydrolysis. ^∗^only observed in a small set of Groups II and III genomes, as identified in Supplementary Table S4.

The genomes of 156 strains of *C. botulinum* Groups I–IV and *C. sporogenes* all contained CLE homologs, with the level of conservation in the predicted protein sequences within each Group >90%, with the exception of SleB3 and YpeB in Group III (89 and 88%, respectively), similar to the variation in GerX subunit sequences (**Figure 1E**). All *C. botulinum* Groups I and III strains carry at least one copy of *sleB*, with a single exception (Supplementary Table S4). *C. botulinum* Group I strain Af84 appears to lack a SleB homolog, and this is associated with a deletion of a ∼159 kb region in the genome when compared to *C. botulinum* Group I strain U21076. This ∼159 kb deletion also includes the SpoVA channel forming proteins. However, there is a second copy of the gene cluster encoding the SpoVA proteins at a different locus. Furthermore, at the site of the deletion, small fragments of *spoVAD* and *spoVAC* are observed. All *C. botulinum* Group I and *C. botulinum* Group III strains also carry a single copy of a *cwlJ* and *ypeB* gene. Recent studies have shown that, at least for *C. botulinum* Group I ATCC 3502, SleB and YpeB are required for optimal germination and that while a gene encoding CwlJ is present, a functional enzyme is not formed (Meaney et al., 2015). These findings are in contrast to *B. subtilis* in which CwlJ plays a prominent role in degrading the spore cortex (Ishikawa et al., 1998; Yi and Setlow, 2010). Furthermore, studies in our lab (unpublished data) revealed that insertional inactivation of *cwlJ* in *C. sporogenes* ATCC15579 resulted in a decrease in germination rate, indicating that CwlJ is functional in this strain. Our bioinformatic analysis also revealed the presence of genes encoding four additional ‘SleB-like’ proteins (tentatively named SleB2, SleB3, SleB4, and SleB5), with at least one example present in each strain of *C. botulinum* Groups I–III (**Figures 3** and **4**; Supplementary Table S4).

Interestingly, *C. botulinum* Group II, which possess the SleC cortex hydrolysis system, also contain *sleB2*, and strains CB11/1-1, 20536 and ATCC17786 contain a second *sleB*-like CDS (encoding SleB4). The latter *sleB*-like gene may, however, be involved in bacterial conjugation as in strains CB11/1-1 and ATCC17786 it is present on a plasmid, situated adjacent to genes predicted to encode the conjugation pilus (Carter et al., 2016). We did not detect genes encoding YpeB or CwlJ homologs in *C. botulinum* Group II genomes. Similarly, *C. botulinum* Group IV encodes a SleC cortex hydrolysis system, and does not encode SleB-like homologs. *C. botulinum* Group II strains also contain a second *sleC*-like CDS (*sleC2*a/b; Supplementary Table S4). Alignment and secondary structural analysis revealed that SleC, which is ∼443 residues long, consists of two domains; a SpoIID/LytB domain plus a peptidoglycan-binding domain. SleC2a/b, annotated as a spore CLE, pre-pro-form, is also a multi-domain protein consisting of a SpoIID/LytB domain and 4–5 peptidoglycan-binding domains. SleC2a is ∼792 residues and SleC2b is ∼698 residues. SleC2b, which has one less peptidoglycan-binding domain in comparison to SleC2a, is largely found in *C. botulinum* Group II type E strains.

SleB2, which is usually annotated in the databases as being ‘SleB-like’ with a similar C-terminus to *B. cereus* SleB, is ∼177 residues and is defined as a cell wall hydrolase. SleB3 is ∼260 residues, consists of two LysM domains, which are involved in peptidoglycan-binding, and is also associated with the Hydrolase_2 superfamily. Both SleB2 and SleB3 CDSs are immediately preceded by an *ydaO* element (except in *C. botulinum* Group II strains). The *ydaO* element is a riboswitch that is often associated with genes responsible for degradation of polysaccharides in *Bacillus* (Block et al., 2010). SleB4 is ∼214 residues, is only found in three *C. botulinum* Group II strains and belongs to the cell wall hydrolase family. SleB5 is only found in one *C. botulinum* Group III strain, has two peptidoglycan-binding domains and is annotated as belonging to the spore cortex-lytic family. It is interesting that strains which use the SleC CLE system also contain these SleB-like proteins and it is tempting to speculate that these proteins may be involved in an alternative cortex hydrolysis pathway. Certainly, the functionality of these *sleB* and *sleC*-like CDSs merits further investigation, including determination of the ligand for the *ydaO* motif.

### A Diagrammatic Germination Model for Spore Germination in *C. botulinum* Groups I–IV

Although the germination mechanisms in *Bacillus* are better understood, significant progress is now being made in understanding germination processes in *Clostridium* (Paredes-Sabja et al., 2011; Kevorkian et al., 2016). Despite this progress, germination mechanisms in *C. botulinum* Groups I–IV are still relatively poorly understood. Based on our findings and on studies in *Bacillus* and *C. perfringens* (Paredes-Sabja et al., 2011) we propose two germination systems for *C. botulinum* spores (**Figure 4**). The first system, which applies to strains of *C. botulinum* Group I (and *C. sporogenes*) and *C. botulinum* Group III, involves the recognition of nutrient germinants by their cognate receptor, followed by Ca^2+^-DPA release through the proposed SpoVA channel (Stage I). In stage II, the CLEs CwlJ and SleB are activated, followed by cortex hydrolysis, membrane and coat degradation, the recommencement of metabolism and eventually cell outgrowth (**Figure 4**). The second germination system, present in strains of *C. botulinum* Group II and *C. botulinum* Group IV, again involves the recognition of nutrient germinants by their cognate receptor, followed by Ca^2+^-DPA release through the proposed SpoVA channel (Stage I). In stage II, the CLE SleC is activated by CspB (a protein only found in Groups II and IV, see Supplementary Tables S2 and S4), followed by cortex hydrolysis, membrane, and coat degradation, the recommencement of metabolism and eventually cell outgrowth (**Figure 4**). These are the first models proposed for spore germination in *C. botulinum* Groups I–IV.

## Conclusion

This work describes the first models to be developed of spore germination in *C. botulinum* Groups I–IV. Of particular interest is the discovery that two different pathways exist which lead to spore germination and subsequently to outgrowth. Spore germination followed the phylogenetic groupings, with germination in *C. botulinum* Groups I and III similar, and subtly different to that in *C. botulinum* Groups II and IV. The bioinformatic comparisons and comparative genomics analyses suggest that it is most likely that the individual GerA/B/C components of the GerX clusters have co-evolved, although we cannot exclude a contribution from recombination and horizontal gene transfer. The next few years may be a fascinating period of discovery, in which the different specific role of each class of GR is uncovered.

## Author Contributions

JB and MP conceived the study. JB, MP, AvV, AC, and FvdB analysed the data. JB, AvV, and FvdB performed the experiments. JB, MP, AC, and AvV wrote the paper. All authors read and approved the final manuscript.

## Conflict of Interest Statement

The authors declare that the research was conducted in the absence of any commercial or financial relationships that could be construed as a potential conflict of interest.

## Footnotes

1 http://www.ncbi.nlm.nih.gov/genome/browse/

2 http://sourceforge.net/projects/ffp-phylogeny/

3 http://tree.bio.ed.ac.uk/software/figtree/

4 http://www.geneious.com
