# Supplementary material for: Diversity of the Germination Apparatus in Clostridium botulinum Groups I, II, III, and IV
Source: Front Microbiol. 2016 Oct 28;7:1702. doi: 10.3389/fmicb.2016.01702 (PMC5083711; doi:10.3389/fmicb.2016.01702)
Supplement: Supplementary file 5 [file Data_Sheet_1.ZIP › Description_files_in_archive.pdf]

Brunt et al, Diversity of the Germination Apparatus in *Clostridium botulinum* Groups I, II, III and IV

Description of files in archive:

Brunt_Fig1A_tree_parsnp.nwk:	Tree used for Figure 1A
Brunt_Fig1B_tree_parsnp.nwk	Tree used for Figure 1B
Brunt_Fig1C_tree_parsnp.nwk	Tree used for Figure 1C
Brunt_Fig1E_GerA_aminoacid_alignment.fasta	Alignment used to generate tree Fig. 1E
Brunt_Fig1E_GerA_aminoacid_alignment_tree.nwk	Tree used for Figure 1E
Brunt_Fig1E_GerB_aminoacid_alignment.fasta	Alignment used to generate tree Fig. 1E
Brunt_Fig1E_GerB_aminoacid_alignment_tree.nwk	Tree used for Figure 1E
Brunt_Fig1E_GerC_aminoacid_alignment.fasta	Alignment used to generate tree Fig. 1E
Brunt_Fig1E_GerC_aminoacid_alignment_tree.nwk	Tree used for Figure 1E
Brunt_Fig3_SleB_aminoacid_alignment.fasta	Alignment used to generate tree Fig. 3
Brunt_Fig3_SleB_aminoacid_alignment_tree.nwk	Tree used for Figure 3
Brunt_FigS1_tree_FFP.nwk	FFP tree from Figure S1
Brunt_FigS1_tree_FFP_bootstrap_consense.nwk	Consense generated bootstrap output for FFP tree used in Figure S1
