# Supplementary material for: Diversity of the Germination Apparatus in Clostridium botulinum Groups I, II, III, and IV
Source: Front Microbiol. 2016 Oct 28;7:1702. doi: 10.3389/fmicb.2016.01702 (PMC5083711; doi:10.3389/fmicb.2016.01702)
Supplement: Supplementary file 6 [file Image_1.PDF]

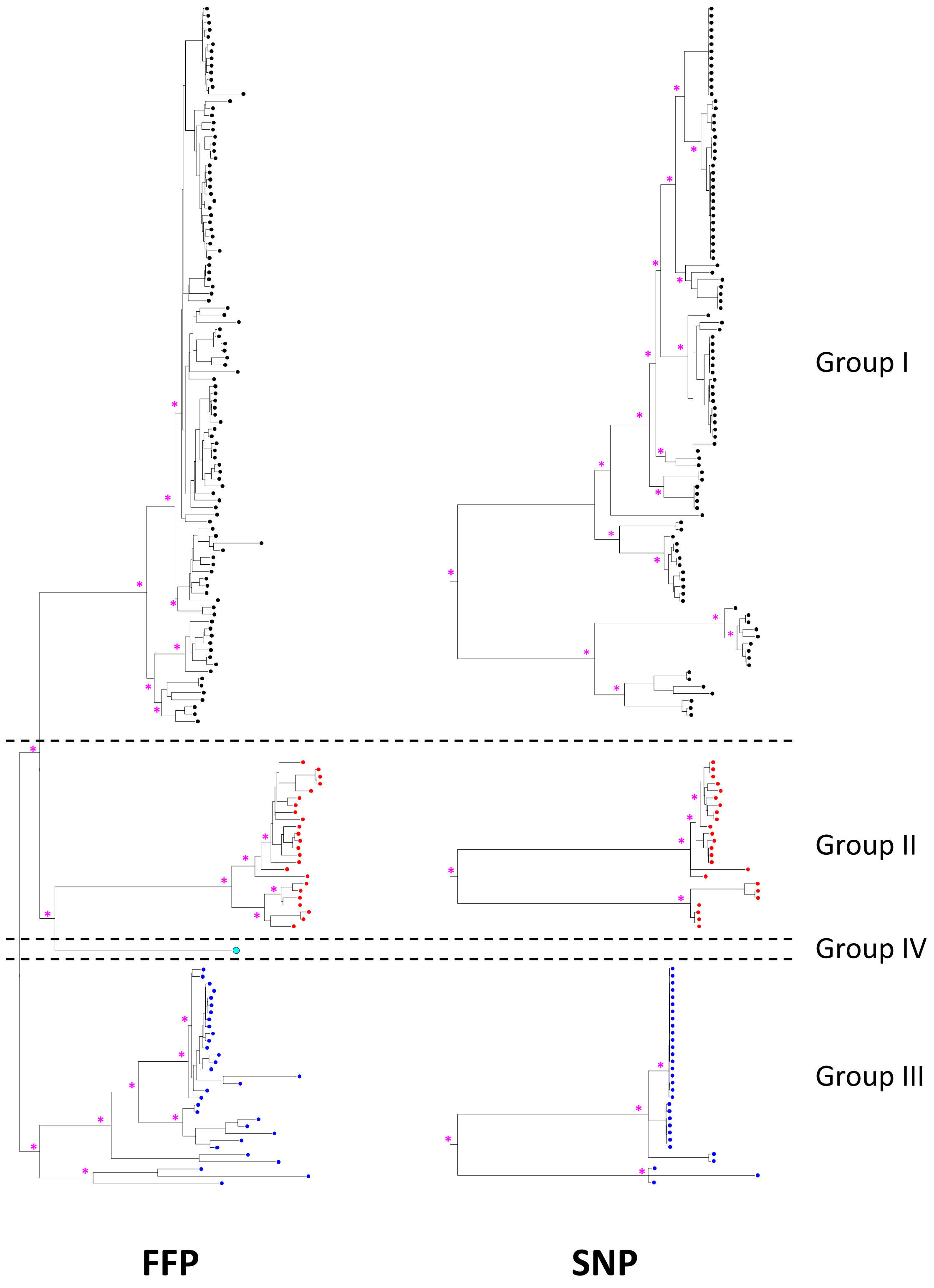

Figure S1. Comparison of core genome SNP analysis and feature frequency profiling (FFP) of *C. botulinum* genomes. Pink asterisks at major branches indicate bootstrap values of >95%, based on 100 replicates (FFP) and the bootstrap values provided in the ParSNP output. Colored dots represent individual genomes from Groups I-IV.
